# BCG cell wall skeleton augments the immunogenicity of dengue nanoparticle vaccines by promoting dendritic cell activation

**DOI:** 10.1371/journal.pone.0337113

**Published:** 2025-11-20

**Authors:** Jitra Limthongkul, Saradee Warit, Panya Sunintaboon, Sukathida Ubol, Tuksin Jearanaiwitayakul

**Affiliations:** 1 Department of Microbiology, Faculty of Science, Mahidol University, Bangkok, Thailand; 2 Industrial Tuberculosis Team, Industrial Medical Molecular Biotechnology Research Group, BIOTEC, National Science and Technology Development Agency, Thailand Science Park, Pathum Thani, Thailand; 3 Department of Chemistry, Faculty of Science, Mahidol University, Salaya, Nakhon Pathom, Thailand; 4 Department of Clinical Pathology, Faculty of Medicine Vajira Hospital, Navamindradhiraj University, Bangkok, Thailand; Universitas Padjadjaran, INDONESIA

## Abstract

Bacillus Calmette-Guérin cell wall skeleton (BCG-CWS) has been shown to enhance vaccine effectiveness and antitumor immunity. In our previous study, we reported that co-administration of BCG-CWS with the encapsidated dengue antigens, UV-inactivated DENV2 and DENV2 NS1, synergistically induced DENV-specific adaptive immune responses in mice. As dendritic cells (DCs) are key immune players that mediate innate and adaptive immunity, we, here, asked how well the response of DCs to this adjuvant aligns with the immune responses elicited *in vivo*. The responses of primary monocyte-derived DCs to BCG-CWS-adjuvanted encapsidated dengue immunogens compared with the unadjuvanted vaccine were investigated. DCs stimulated by BCG-CWS and the dengue nanoparticle vaccine exhibited a superior response. This response correlated well with the stronger immune response observed in mice. This was evidenced by the marked elevation in expression levels of DC activation markers, such as CD80, CD83, CD86, and HLA-DR, and various innate immune cytokines. Additionally, this adjuvant markedly elevated the expression levels of miRNAs related to DC function, such as miR-146a, miR-147, miR-223, and miR-155. These immune components could suppress DENV2 multiplication in bystander skin cells. BCG-CWS exerted an adjuvant effect on DC responses by enhancing antigen-processing activity and activating several innate immune cytokines and immune-related miRNAs.

## Introduction

Dengue is the most prevalent arboviral disease in the world and is caused by the dengue virus (DENV). DENV consists of four distinct serotypes (DENV1–4), which co-circulate in tropical and subtropical regions. DENV is an enveloped virus, and its genome contains positive-sense (+) single-stranded RNA encoding a single polyprotein of three structural proteins, including C, prM, and E, and seven non-structural (NS) proteins, including NS1, NS2A, NS2B, NS3, NS4A, NS4B, and NS5 [1,2]. DENV infects nearly 390 million people annually, with approximately one-quarter of these infections being symptomatic [3]. Approximately 2%–15% of symptomatic dengue infections can progress to severe disease, which is characterized by increased microvascular permeability and shock syndrome [4]. Management is primarily supportive due to the lack of specific antiviral treatment for dengue.

Over the past decades, global efforts have been made to develop effective vaccines. However, the concurrent existence of all four serotypes of DENV impedes progress. Sequential infection with heterotypic serotypes may cause severe dengue through a mechanism known as antibody-dependent enhancement [5,6]. As of today, two vaccines, Dengvaxia and Qdenga, have been commercialized for use in some countries [7]. Both are tetravalent live attenuated formulations. However, the efficacy of Dengvaxia has been complicated by antigenic interference between the serotypes, resulting in an imbalance of stimulated immune responses against each serotype [8]. Furthermore, the vaccine construct lacks DENV NS genes, limiting its ability to trigger cellular responses [9]. The sensitized DENV immune responses elicited by this vaccine are associated with a higher risk of hospitalization and severe illness upon the occurrence of natural infection [10,11]. On the contrary, Qdenga has shown high efficacy in preventing hospitalization and asymptomatic dengue infection during the initial phase of vaccination [12,13]. However, studies on the long-term efficacy and safety of Qdenga are currently underway [14].

Non-replicating vaccines hold great promise because they are not subjected to viral interference issues due to dose adjustment [15]. However, these vaccines often exhibit limited immunogenicity. Thus, these vaccines require potent adjuvants and/or delivery systems to augment their efficacy. Adjuvants can enhance immune responsiveness through diverse mechanisms. For example, alum, the most commonly used adjuvant in human vaccines, enhances vaccine effectiveness by forming an antigen depot [16,17]. This adjuvant effect not only prolongs the retention time of antigens at the injection site but also increases the uptake of antigens by immune cells [18]. Alum is a potent inducer of antibody production [19]. Unfortunately, it poorly induces Th-1 type and CD8^+^ T-cell immune responses [20–22]. Therefore, the triggered response may be less effective against viruses and other intracellular pathogens. We previously reported that the addition of the Bacillus Calmette-Guérin cell wall skeleton (BCG-CWS) increased the immunogenicity of the DENV2 encapsidated immunogens, DENV2 NS1 and UV-inactivated DENV2 (UV-DENV2) [23]. This adjuvant coordinated with the delivery system enhances the Fc effector function of the produced antibodies and strongly stimulates polyfunctional T cells [23]. However, the mechanisms underlying the enhanced adjuvanticity of BCG-CWS in the dengue nanoparticle vaccine have not been elucidated.

As dendritic cells (DCs) represent a major link between innate and adaptive immunity and are abundantly present at vaccine injection sites [24], we aimed to address the potential mechanism of BCG-CWS enhanced immunogenicity of dengue immunogens at the cellular and molecular levels in monocyte-derived dendritic cells (moDCs), an *ex vivo* experiment. Therefore, the adjuvant effect of BCG-CWS on the maturation and activation of immature moDCs in the presence of dengue nanoparticle vaccines was investigated. We here demonstrated that BCG-CWS could provide collaborative adjuvant function with *N,N,N*-trimethyl chitosan nanoparticles (TMC NPs). The combination of BCG-CWS and nanodelivery system synergistically induced the costimulatory signals, innate immune cytokine secretion and immune-related-miRNA expression. This ultimately enhanced more intense moDC maturation. Interestingly, the induced innate response also triggered the anti-DENV response in the bystander human dermal fibroblast.

## Materials and methods

### Ethics statement

The study involving human peripheral blood mononuclear cells (PBMCs) was conducted in accordance with the Declaration of Helsinki and approved by the Ethical Review Board of the Faculty of Medicine, Vajira Hospital, Navamindradhiraj University (COE: 26/2024 X). PBMCs were isolated within 6 h after collection from buffy coats of healthy blood donors. This blood component was provided by a blood bank service at Vajira Hospital during July 2024 – January 2025. The need for informed consent was waived by the ethics committee due to that the provision of buffy coats was made anonymously without indication of donor personal data.

### Cells and viruses

The moDCs used in this experiment were generated from purified monocytes from PBMCs of healthy donors as previously described [25]. Vero cells were grown in Minimum Essential Media (Gibco, Thermo Fisher Scientific, Inc., Amarillo, TX, USA) supplemented with 10% fetal bovine serum (Gibco). Human dermal fibroblasts (HDFs, Lonza, Basel, Switzerland) were cultivated in Dulbecco’s modified Eagle’s medium (Gibco) containing 10% fetal bovine serum. DENV2 (strain 16681) was propagated in C6/36 cells, aliquoted, and stored at −80°C. Virus titers in Vero cells were quantified using a plaque assay.

### Preparation of dengue nanoparticles and BCG-CWS

UV-inactivated DENV2 *N,N,N*-trimethyl chitosan nanoparticles (UV-DENV2 TMC NPs) and NS1-TMC NPs were obtained from Jearanaiwitayakul et al. These immunogens and BCG-CWS were prepared as previously described [23].

### Uptake of BCG-CWS or BCG-CWS-adjuvanted dengue nanovaccine by moDCs

MoDCs were incubated with BCG-CWS (7.5 μg/mL), empty TMC NPs, or encapsidated dengue immunogens; NS1-TMC NPs or UV-DENV2 TMC NPs (100 µg/mL) in the presence or absence of BCG-CWS (7.5 μg/mL) at 37°C with 5% CO_2_ for 24 h. After incubation, the treated cells were washed, fixed, and permeabilized with Cytofix/Cytoperm™ solution kit (BD biosciences, San Diego, CA, USA). Intracellular NS1, UV-DENV2, and BCG-CWS were stained with 6 × His Tag monoclonal antibody (Invitrogen, Carlsbad, CA, USA), mouse anti-DENV2 monoclonal antibody (3H2) (GeneTex, Irvine, CA, USA), and rabbit anti-mycobacterial cell wall polyclonal antibody (Abnova, Tebu, France), respectively. Alexa Fluor™ 488-conjugated anti-mouse antibody and Alexa Fluor™ 568-conjugated anti-rabbit antibody (Invitrogen) were used as secondary antibodies. Stained cultures were visualized and captured using BioTek Cytation 7 (Agilent, USA).

### Immunostimulatory effects of BCG-CWS and dengue nanoparticle vaccines on moDC maturation

The culture of moDCs was incubated with BCG-CWS (7.5 μg/mL), empty TMC NPs, encapsidated immunogens (NS1-TMC NPs or UV-DENV2 TMC NPs), or encapsidated immunogens adjuvanted with BCG-CWS. The treated medium was used as a negative control. The cells were harvested at 24 and 48 h after treatment for surface staining with antibodies specific to DC maturation markers (CD80, CD83, CD86, and HLA-DR). The frequency and mean fluorescent intensity (MFI) of the stained cells were determined using flow cytometry (CytoFLEX, Beckman Coulter, Indianapolis, IN, USA). The cultured supernatants were collected and analyzed for quantitation of cytokine and chemokine levels using a Bio-Plex human cytokine assay kit (Bio-Rad Laboratories, Hercules, CA, USA) following the manufacturer’s instructions. The amounts of secreted type I interferon (IFN), IFN-α (Cusabio, Hubei, China), and interleukin (IL)-27 (R&D Systems, Minneapolis, MN, USA) were quantified separately using ELISA kits.

### MicroRNA (miRNA) expression analysis

Treated cells were harvested 3 and 24 h after treatment and were subjected to miRNA detection as previously described [26]. In brief, purified RNA (10 ng) was converted into cDNA using specific primers and the TaqMan™ MicroRNA Reverse Transcription Kit (Applied Biosystems, Foster City, CA, USA). The miRNA expression level was monitored by qPCR using TaqMan™ MicroRNA assays (Applied Biosystems) following the manufacturer’s instructions. Data were analyzed using the comparative CT (ΔΔCT) method to calculate the relative quantitation of target miRNA expression as previously described [26].

### Anti-DENV activity of supernatants of moDCs treated with BCG-CWS-adjuvanted dengue nanovaccine

The supernatants from the moDC-treated cells were spun down at 10,000 × g for 10 min to remove the unbound nanoparticles and BCG-CWS before being added onto the monolayer of the HDFs to evaluate the antiviral effects of the mediators secreted from the treated moDCs on the skin cells. The treatment was conducted at 37°C under 5% CO_2_ for 24 h. After incubation, the cells were washed twice with phosphate-buffered saline and then challenged with DENV2 at a multiplicity of infection of 10. The cells were harvested 24 h after infection for antibody staining with a mouse anti-flavivirus antibody (4G2). The stained cells were analyzed using flow cytometry. Aliquots of the supernatant were harvested, and the titer of infectious particles was measured using a plaque assay as previously described [26]. Cells treated with fresh media were used in this experiment as mock-treated cultures.

### Statistical analysis

Data analyses were performed using Student’s *t*-test for comparisons between two groups and one-way ANOVA followed by Bonferroni’s post hoc comparison tests for comparisons between multiple groups. A *p*-value <0.05 was considered statistically significant.

## Results

### Uptake of dengue nanoparticle vaccine and BCG-CWS by moDCs

**Fig 1:**
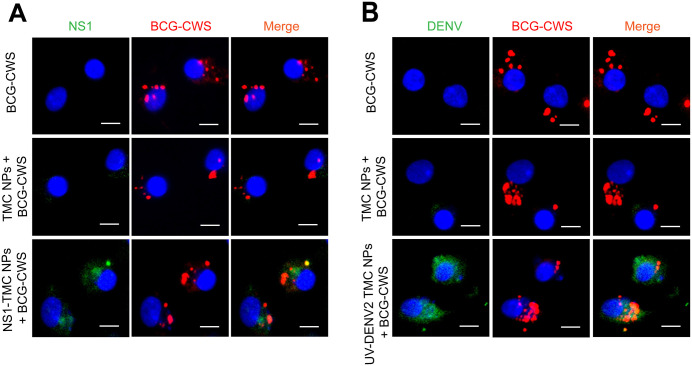
Uptake of BCG-CWS and encapsidated NS1 or DENV2 antigens by moDCs. MoDCs were treated with BCG-CWS alone, empty TMC NPs, NS1-TMC NPs (100 µg/mL), or UV-DENV2 TMC NPs (100 µg/mL) in the presence of BCG-CWS (7.5 µg/mL) for 24 h. The treated cells were intracellularly stained with mouse anti-His-tag antibody **(A)**, anti-DENV2 E protein antibody (3H5) **(B)**, or anti-mycobacterial cell wall antibody. The stained cells were imaged using a fluorescence microscope. A representative image of two independent experiments is shown. Scale bar = 8 µm.

Whether BCG-CWS and the encapsidated immunogen could be taken up by moDCs was investigated, and the cells were cultured with a mixture of NS1-TMC NPs or UV-DENV2 TMC NPs and BCG-CWS. At 24 h after treatment, intracellular immunogens and BCG-CWS were detected using immunofluorescent staining. The analysis revealed that NS1 or DENV2 antigens colocalized with BCG-CWS (Fig 1A and 1B). This finding indicates that encapsidated immunogens and BCG-CWS are simultaneously internalized by moDCs.

### BCG-CWS strongly stimulated the expression of maturation markers on moDCs

DCs play an important role in shaping the adaptive immune system through maturation and activation. Therefore, the immunogenic enhancement of BCG-CWS on moDC maturation was investigated. The culture of moDCs was incubated with empty TMC NPs, NS1-TMC NPs or UV-DENV2 TMC NPs with or without BCG-CWS for 48 h. The cell viability of moDCs was assessed after exposure to the tested vaccines. All treatment conditions had no cellular toxic effects on moDCs (S1 Fig).

**Fig 2:**
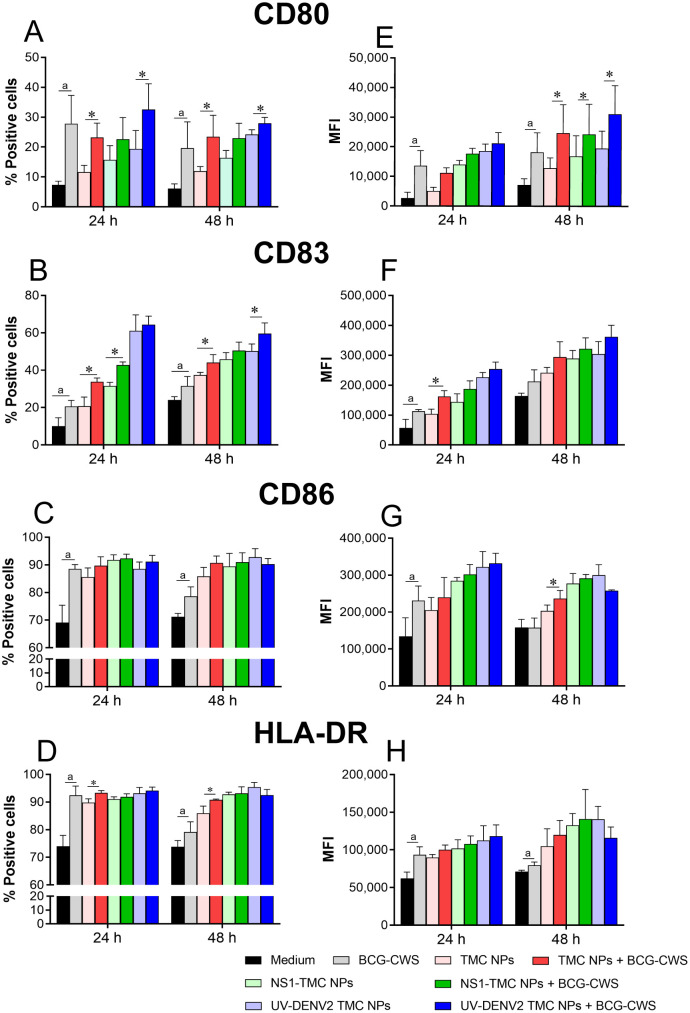
BCG-CWS cooperation with TMC NPs, NS1-TMC NPs, or UV-DENV2 TMC NPs enhanced moDC maturation. The culture of moDCs was incubated with BCG-CWS (7.5 µg/mL), TMC NPs (100 µg/mL), UV-DENV2 TMC NPs, or NS1-TMC NPs (100 µg/mL containing 15 µg/ml of immunogens) in the presence or absence of BCG-CWS (7.5 µg/mL). After incubation, the treated cells were harvested, stained with antibodies specific to DC activation markers, and analyzed for the percentages and expression levels of CD80 **(A, E)**, CD83 **(B, F)**, CD86 **(C, G)**, and HLR-DR **(D, H)** by flow cytometry. Data are presented as mean ± SD (n = 3). ^a^Significant difference between BCG-CWS and the medium. *Significant differences between encapsidated immunogens and encapsidated immunogens supplemented with BCG-CWS (*p* < 0.05).

The maturation markers of DCs, including CD80, CD83, CD86, and HLA-DR, were monitored using flow cytometry. As shown in Fig 2A, BCG-CWS, by itself, upregulated the expression of CD80 on treated moDCs compared with the medium or TMC NPs treatment. Of note, empty TMC NPs slightly upregulated the CD80 expression. Moreover, treatment with the mixture of TMC NPs and BCG-CWS did not reveal the synergistic effect of these two compounds on CD80 expression (Fig 2A and 2E). However, no significant difference in the expression of CD86 and HLA-DR was observed between treatments with or without BCG-CWS (Fig 2C, 2D, 2G, and 2H). This may be because treatment with BCG-CWS alone can drive the expression of CD86 and HLA-DR to the maximum level. Among the activation markers tested, CD83 has been recognized as a definitive marker for mature moDCs [27,28]. We revealed, here, that moDCs increased expression of CD83 on surface upon treatment with BCG-CWS or TMC NPs (Fig 2B and 2F). Interestingly, the CD83 expression was more enhanced following co-treatment with TMC NPs and BCG-CWS. These findings suggest that the delivery system and BCG-CWS may exert a synergistic effect on CD83 expression. Whether both of them will synergize on the moDCs maturation requires further investigation.

### Cytokine/chemokine production by moDCs in response to BCG-CWS-adjuvanted UV-DENV2 or NS1-TMC NPs

**Fig 3:**
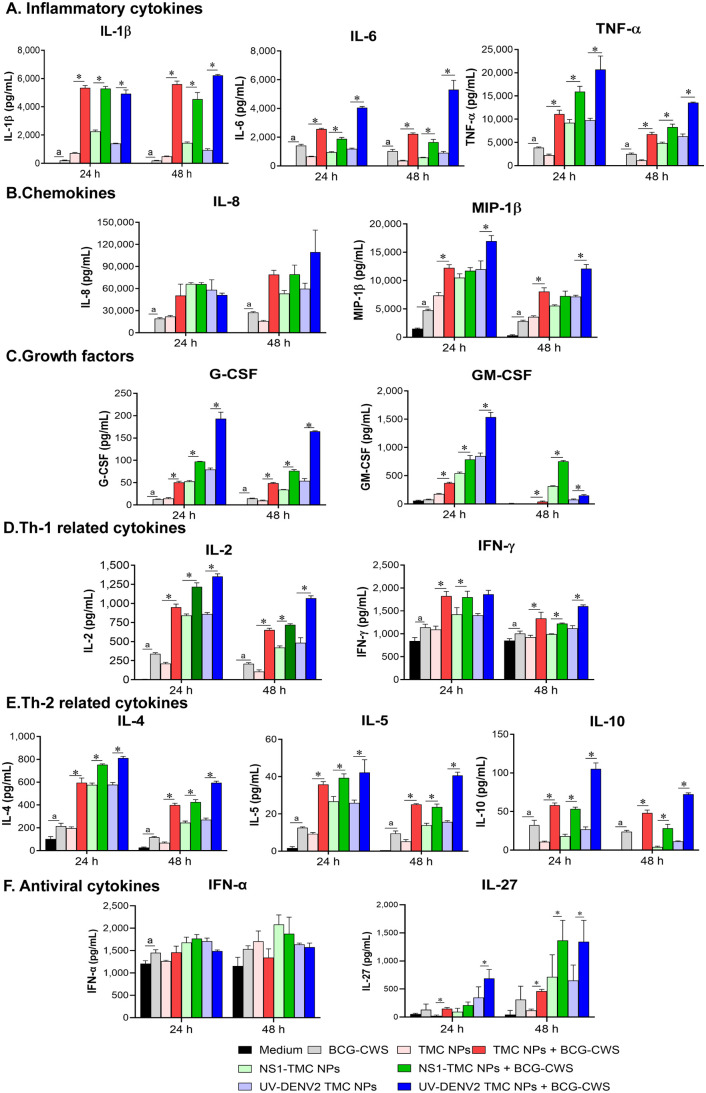
Cytokine/chemokine response of moDCs to BCG-CWS-adjuvanted NS1-TMC NPs or UV-DENV2 TMC NPs. Cells were treated with TMC NPs, NS1-TMC NPs, or UV-DENV2 TMC NPs alone (100 µg/mL containing 15 µg/mL of immunogens) or with BCG-CWS (7.5 µg/mL). The cultured supernatant was collected at 24 and 48 h after treatment. The secreted cytokines/chemokines, including inflammatory cytokines **(A)**, chemokines **(B)**, growth factors **(C)**, Th-1-related cytokines **(D)**, Th-2-related cytokines **(E)**, and antiviral cytokines **(F)**, were measured using the Bio-Plex assay. Data are presented as mean ± SD from three different donors. ^a^Significant difference between BCG-CWS and the medium. *Significant differences between encapsidated immunogens alone and encapsidated immunogens supplemented with BCG-CWS (*p* < 0.05).

The production of cytokines and chemokines is one of the maturation processes of DCs when encountered with foreign antigens. Therefore, the levels of cytokine/chemokine in the culture medium of vaccine-treated moDCs were monitored using a Bioplex-based assay to confirm the maturation of moDCs by encapsidated immunogen plus BCG-CWS. MoDCs strongly responded to treatment with all tested vaccine formulations by upregulating proinflammatory cytokine responses (IL-1β, IL-6, and TNF-α) (Fig 3A). The induction of these cytokines was observed within 24 h of treatment and maintained through the end of our experiments, 48 h after treatment. BCG-CWS cooperated with empty TMC NPs, UV-DENV2 TMC NPs, or NS1-TMC NPs in escalating the levels of proinflammatory cytokine production compared with those stimulations with empty TMC NPs, UV-DENV2 TMC NPs, or NS1-TMC NPs alone (Fig 3A). A similar pattern of results was observed for chemokines (IL-8 and MIP-1β), growth factors (G-CSF and GM-CSF), Th-1-related cytokines (IL-2 and IFN-γ), and Th-2-related cytokines (IL-4, IL-5, and IL-10) in which empty TMC NPs, UV-DENV2 TMC NPs, or NS1-TMC NPs accompanied by BCG-CWS significantly triggered these groups of cytokine responses compared with empty TMC NPs or encapsidated immunogens treated alone (Fig 3B–3E). Additionally, BCG-CWS increased the production of these cytokines from moDC cells compared with the placebo treatment (Fig 3).

The levels of cytokines involved in antiviral responses were measured. Secretion of IFN-α and IL-27 was significantly upregulated in moDCs stimulated by BCG-CWS, TMC NPs, or encapsidated immunogens in the presence or absence of BCG-CWS. Unfortunately, the level of IFN-α produced by moDCs treated with BCG-CWS and encapsidated immunogen was comparable to that of moDCs stimulated by unadjuvanted immunogen counterparts (Fig 3F). Conversely, the addition of BCG-CWS to either empty TMC NPs or encapsidated immunogens stimulated a significant increase in IL-27 levels compared with the vaccine groups without the BCG-CWS adjuvant (Fig 3F). Of note, we observed that 12 out of 14 cytokines were significantly upregulated in moDCs treated with BCG-CWS and TMC/encapsidated immunogens at 24 h or 48 h post-treatment (Fig 3). This consistent upregulation of the majority of detected cytokines suggests that BCG-CWS works with the TMC delivery system/encapsidated immunogens to drive moDC maturation.

### BCG-CWS enhances the expression of miRNAs related to moDC maturation and anti-DENV responses

**Fig 4:**
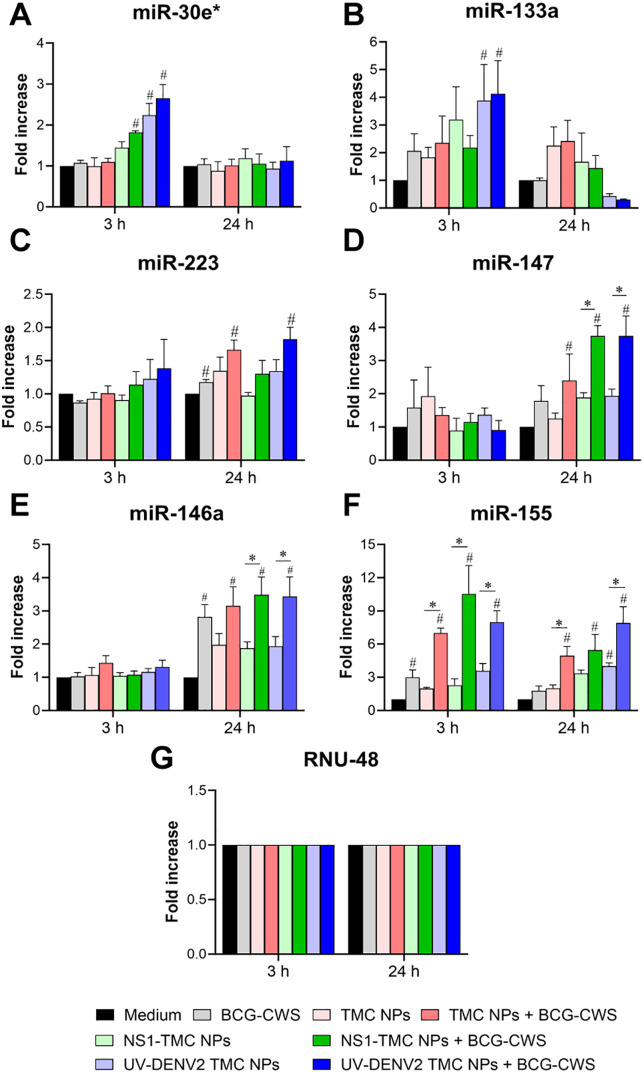
MoDCs exposed to BCG-CWS-adjuvanted dengue nanospheres strongly upregulated miRNAs associated with moDC maturation and anti-DENV response. MoDCs were incubated with BCG-CWS or a mixture of BCG-CWS and dengue nanoparticles. The stimulated cells were harvested 3 and 24 h after treatment. Total miRNAs were isolated and analyzed for the expression levels of miR-30e* **(A)**, miR-133a **(B)**, miR-223 **(C)**, miR-147 **(D)**, miR-146a **(E)**, and miR-155 **(F)** using qRT-PCR. **(G)** RNU48 was used as a reference miRNA. Data are presented as mean ± SEM (n = 3). ^#^Significant differences between vaccine- or adjuvant-treated groups and medium-treated control. *Significant differences between the BCG-CWS-adjuvanted encapsidated immunogen and the immunogens without BCG-CWS.

MiRNAs are non-coding endogenous RNAs that regulate the responses associated with innate and adaptive immunity. This class of small non-coding RNAs manipulates the host transcriptome by binding to the complementary 3′-UTR of mRNA and promoting mRNA degradation, thereby inhibiting protein synthesis [29]. MiRNAs modulate gene expression within producing or neighboring cells via exosome-containing miRNAs [30]. Therefore, the potential effect of BCG-CWS on the immunogenicity of dengue nanospheres at the miRNA level was explored. MoDCs were incubated with BCG-CWS, empty TMC NPs, or encapsidated immunogens with or without BCG-CWS. The treated cells were harvested 3 and 24 h after treatment to monitor the expression of miRNAs related to immune and anti-DENV responses using qRT-PCR. Treatment of encapsidated immunogens in the presence or absence of BCG-CWS transiently upregulated miR-30e* expression within 3 h after treatment (Fig 4A). The expression rapidly decreased to a basal level after 24 h. A similar trend of results was observed for miR-133a expression. Among the tested stimuli, encapsidated immunogens accompanied by BCG-CWS exerted the strongest inducer of this miRNA expression (Fig 4B). Additionally, increased miR-223 expression was detected. At 24 h after treatment, moDCs exposed to empty NPs or UV-DENV2 TMC NPs adjuvanted with BCG-CWS expressed a significant miR-223 level compared with the medium-treated control (Fig 4C). Furthermore, altered expression of miR-146a, miR-147, and miR-155 was detected (Fig 4D–4F.). For miR-146a and miR-147 expression, BCG-CWS-adjuvanted empty TMC NPs or encapsidated immunogens significantly upregulated higher levels of expression of these miRNAs at 24 h of treatment compared with their counterparts without BCG-CWS (Fig 4D and 4E). Conversely, the level of miR-155 expression stimulated by these stimuli was significantly elevated as early as 3 h of stimulation and sustained through the end of the experiment at 24 h after treatment (Fig 4F).

The *ex vivo* results showed that BCG-CWS increased the immunogenicity of encapsidated immunogens by enhancing the production of innate immune cytokines/chemokines and the expression of immune response-related miRNAs.

### Soluble mediators secreted from treated moDCs induce DENV2 resistance in human skin cells

**Fig 5:**
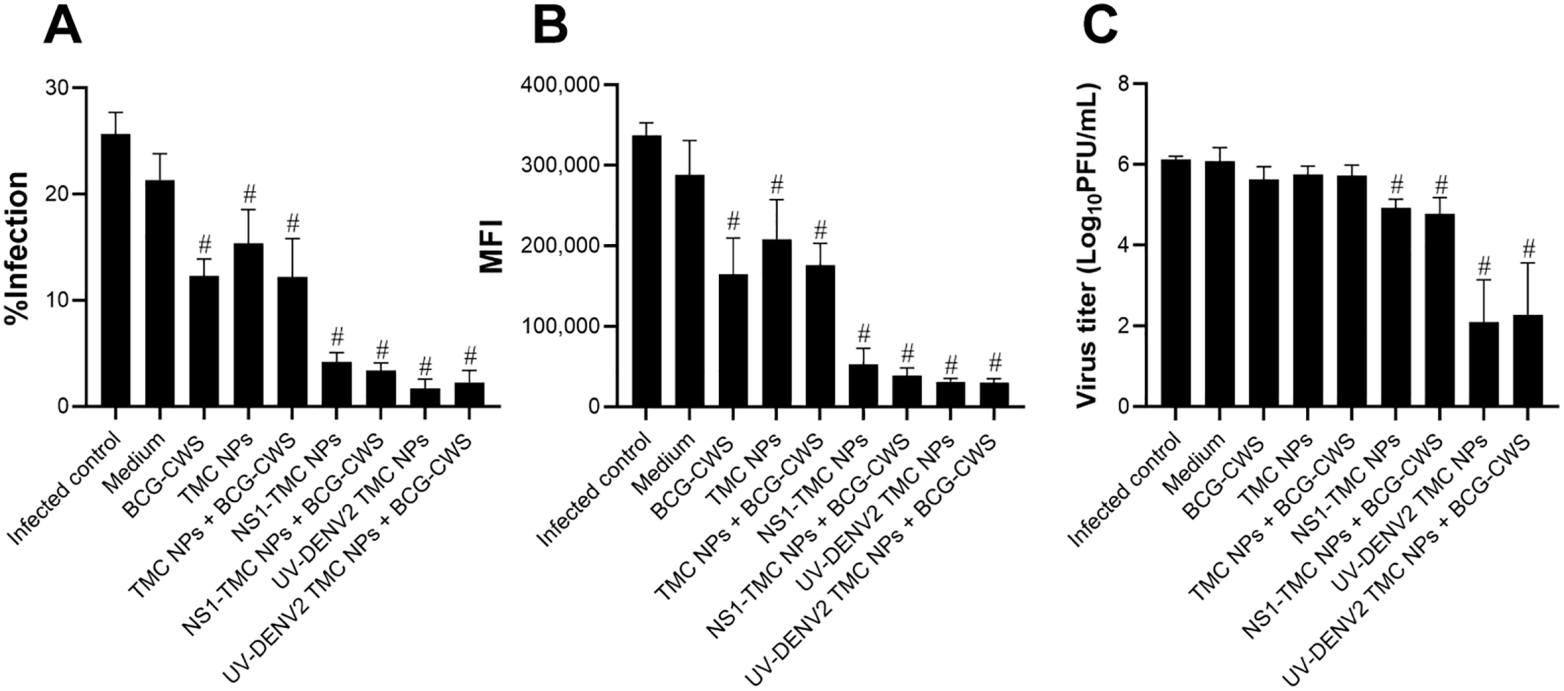
Anti-dengue activity of moDCs supernatant treated with BCG-CWS-adjuvanted nanovaccine in human skin cells. HDFs were cultured with the supernatant derived from moDCs treated with BCG-CWS alone or with TMC NPs, NS1-TMC NPs, or UV-DENV2 TMC NPs for 24 h. After incubation, the treated HDFs were infected with DENV2 strain 16681 at a multiplicity of infection of 10. The cells were harvested and intracellularly stained with an anti-flavivirus antibody (4G2) at 24 h after infection. The percentage of DENV2-positive cells **(A)** and MFI **(B)** were determined by flow cytometry. The virus titer of DENV2 in cultured supernatants was measured using plaque assay **(C)**. Data are presented as mean ± SD (n = 3). ^#^Significant differences between the vaccine- or adjuvant-treated groups and the medium-treated control (*p* < 0.05).

Exposure to the virus strongly triggers an antiviral response in DCs upon DENV recognition [31,32]. These activated DCs cross-talk and control early DENV infection of neighboring skin cells, such as dermal fibroblasts [33]. Therefore, whether antiviral mediators secreted from treated moDCs could stimulate an antiviral state of bystander skin cells was investigated. Primary HDFs were stimulated by the supernatant obtained from treated moDCs for 24 h before being infected with DENV2. Viral multiplication in the treated cells was detected using flow cytometry and plaque assay. The stimulation of HDFs with supernatants of moDCs treated with empty TMC NPs or encapsidated immunogens alone or with BCG-CWS resulted in an efficient reduction of DENV infection (Fig 5A and 5B). This result corresponded to the viral titer in which treatment of the culture supernatant from moDCs stimulated by these stimuli significantly suppressed viral production by >10-fold compared with the medium-treated group (Fig 5C). However, no significant difference in anti-DENV2 activity was observed (Fig 5A–5C). Interestingly, the supernatant treated with BCG-CWS alone significantly reduced DENV2 infection (Fig 5A and 5B). These findings indicate that soluble mediators activated by BCG-CWS and encapsidated immunogens potently drive HDFs into the anti-DENV state.

## Discussion

The adjuvant function of BCG-CWS for cancer immunotherapy has been extensively studied. However, its potential as a vaccine adjuvant against infectious diseases has not been fully explored. BCG-CWS consists of immunologically active components, including mycolic acid and peptidoglycan complex covalently linked to arabinogalactan [34]. These microbial compounds can be recognized by TLR2 and TLR4 in DCs [34,35]. This interaction further stimulates DC maturation in a MyD88-dependent fashion, enhancing the antigen-specific immune response [35,36]. Our previous study showed that BCG-CWS is an effective adjuvant for dengue nanoparticle vaccines [23]. The adjuvant properties of BCG-CWS are related to the improved neutralizing antibody responses through enhanced Fc effector function and elevated cellular Th-1/Th-2 responses [23]. Therefore, moDCs were stimulated by BCG-CWS-adjuvanted dengue nanoparticles, and their responses were evaluated by measuring the expression of their activation markers and innate immune mediators to elucidate insights into the adjuvanticity of BCG-CWS.

The results showed that the TMC NP-loaded dengue antigens combined with BCG-CWS were efficiently engulfed and showed good biocompatibility with moDCs. This finding is consistent with that previously observed in our *in vivo* setting [23], which supports the favorable safety profile of this vaccine mixture. In addition to safety profiling, the results showed that the magnitude of moDC responses to administered antigens increased synergistically upon adjuvanation with BCG-CWS. This was illustrated by the marked upregulation of the expression of maturation markers, including CD80, CD83, CD86, and HLA-DR, and various innate immune cytokines/chemokines. This *ex vivo* observation was positively correlated with robust immune responses observed in immunized mice [23]. These activating markers, along with innate immune mediators, may play an indispensable role in establishing effective adaptive immunity. For example, IFN-γ accompanied with IL-12 promotes the differentiation of naive T cells into Th-1, Th-1/Th-17 subsets, and CD8 ⁺ T cells [37–39], whereas IL-4 and IL-6 are known as Th-2 promoting cytokines shaping Th-2 cell conversion and antibody production, respectively [40,41]. The increased levels of these innate immune components observed in this study indicate a potential mechanism underlying immunogenic enhancement by the BCG-CWS adjuvant.

Aluminum is well recognized as the standard adjuvant for multiple types of licensed vaccines such as anthrax, diphtheria, hepatitis A and B, Japanese encephalitis and pneumococcal disease [42]. Despite its long history of use, its mechanism of action remains unclear. It is believed that aluminum salt-based adjuvants exhibit their activity and potency through a depot effect or deposit at the inoculation site. This prolongs antigen release in a controlled time-based manner [43]. It has also been reported that alum-based adjuvants may cause tissue damage at the injection site. This subsequently triggers the release of damage-associated molecular patterns (DAMPs), then recruiting immune cells to that area [43,44]. Upon administration, alum is highly effective at inducing a strong humoral response, but it is poor at provoking cytotoxic T lymphocyte (CTL) response. The mechanisms underlying this selective enhancement of humoral immunity are still not fully understood. This may be due to that alum predominantly induces Th-2 response [45]. Interestingly, our *ex vivo* and *in vivo* results revealed that BCG-CWS adjuvant confers a robust induction of both Th-1 and Th-2 responses. This was shown by the upregulation of moDC-derived Th-1/Th-2 cytokines (Fig 3) and the production of IgG1/IgG2a antibodies [23]. These findings suggest that BCG-CWS may elicit a more balanced Th-1 and Th-2 response compared to alum adjuvant.

Many miRNAs have been reported to positively and negatively regulate DC differentiation and function [46]. For example, miR-155 is a master regulator affecting the maturation state of DCs. Mechanistically, miR-155 targets the 3′UTR of c-Fos, a negative regulator of DC function [47]. DCs deficient in miR-155 downregulate the expression of costimulatory molecules (MHC II, CD83, CD86, and CD40) and proinflammatory cytokines (IL-12 and TNF-α), resulting in the deprivation of antigen-specific T-cell responses [47]. These findings suggest that miR-155 promotes the activating state of DCs. miR-146a is another miRNA that exerts an inverse impact on DC activation. This miRNA inhibits TLR-mediated nuclear factor kB activity by targeting IRAK-1 and TRAF-6 [48]. This interaction downregulates the activation state of DCs, resulting in reduced production of proinflammatory mediators [48]. In the present study, we showed that BCG-CWS significantly upregulated the expression of miRNAs related to proinflammatory and anti-inflammatory responses. These findings are consistent with those of cytokine profiling, suggesting that BCG-CWS not only potently drives moDC maturation but also helps fine-tune the inflammatory response at the injection site. Collectively, these findings suggest that BCG-CWS orchestrates moDC function by enhancing cytokine/chemokine production and regulating the expression of immune-associated miRNAs.

In addition to their ability to augment the immunogenicity of encapsidated immunogens, both BCG-CWS and encapsidated immunogens can induce innate anti-DENV responses. This was evidenced by the significant reduction in DENV multiplication in HDFs stimulated by the moDC supernatant. Based on the current analysis of altered innate responses, we hypothesized that two groups of innate immune components participate in the defense against DENV. The first group consists of antiviral cytokines (IFNs and IL-27). Type I IFN has long been recognized as a strong DENV antagonist [49], and IL-27 has recently been explored for its anti-DENV activity [50]. Both cytokines control DENV replication by upregulating the expression of interferon-stimulated genes [51]. The other group potentially involved in DENV containment is the miRNAs associated with the anti-DENV response. These included miR-30e*, miR-133a, miR-155 and miR-233, all of which were robustly upregulated following stimulation of the BCG-CWS-adjuvanted DENV nanosphere. miR-133a directly affects DENV RNA replication by targeting a sequence in the 3’UTR of the DENV genome [52], whereas miR-223 modulates cellular function by targeting the 3’UTR of the STMN1 gene, which encodes a microtubule instability protein [53]. The downregulation of STMN1 expression has been reported to substantially hinder DENV replication [53]. Furthermore, miR-30e* and miR-155 exert antiviral effects by targeting SOCS1, a negative regulator of type I IFN signaling, thereby enhancing the production of antiviral interferon responses [54,55]. Given that TMC NPs and BCG-CWS had a synergistic effect in inducing the innate antiviral response, the magnitude of anti-DENV suppression showed no statistical differences between encapsidated immunogen in the absence and presence of BCG-CWS. This uneven result may be due to the fact that TMC-loaded dengue antigens may possess superior anti-DENV activity, whereas BCG-CWS partially did so. Because type I IFNs are known as the most potent antiviral molecules, we hypothesized that type I IFNs are likely the key effectors of DENV suppression in our setting. This hypothesis was supported by the finding that the level of IFN-α negatively correlated with the level of viral multiplication as shown in Fig 3.

This current work presented two main limitations. First, there is a lack of additional experiments, such as functional validation assays to confirm the roles of the identified molecules in the pathways. Second, the use of a minimal sample size may affect the statistical power of the findings. Thus, the relevant functional assay with larger sample sizes deserves further study.

### Conclusion

**Fig 6:**
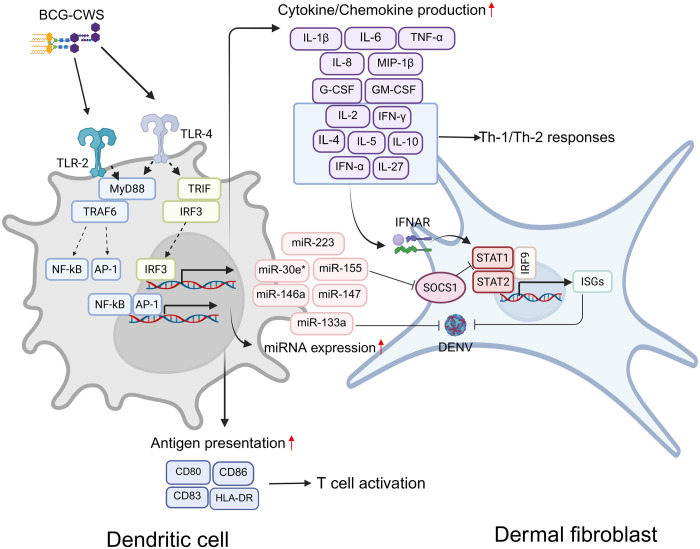
The potential working mechanisms of how BCG-CWS enhances the immunogenicity of dengue nanoparticle vaccines. Upon binding to TLR-2 and TLR-4 on moDCs, BCG-CWS potentially activates downstream transcription factors via a MyD88-dependent fashion, including NF-κB and activator protein 1 (AP1). These transcription factors upregulate the expression of genes involved in antigen presentation (e.g., CD80, CD83, CD86 and HLA-DR) and cytokine/chemokine production. MHC and costimulatory molecules participate in initiating T cell activation, while moDC-derived cytokines related to Th-1/Th-2 responses help shape the differentiation of specific T helper cell subsets and influence IgG subclass switching. Furthermore, BCG-CWS may stimulate MyD88-independent (TRIF-dependent) mechanism. This signaling induces activation of IRF3, leading to production of type I interferon. This cytokine can promote cell-intrinsic antiviral states of neighboring cells. The mechanism underlying viral suppression may be associated with the expression of antiviral proteins encoded by interferon-stimulated genes (ISGs), potentially via STAT1/STAT2/IRF9 signaling axis. In addition, BCG-CWS significantly upregulates miRNA expression, such as miR-155 and miR-146a. These miRNAs are responsible not only for fine-tuning inflammation response but also for regulating innate antiviral responses. Created in BioRender. Limthongkul, J. (2025) https://BioRender.com/8zxymdr.

This study demonstrated the adjuvant activity of BCG-CWS for enhancing the immunogenicity of dengue nanovaccines. The potential mechanisms by which BCG-CWS enhances moDC function are illustrated in Fig 6. These include promoting antigen presentation, stimulating the production of innate immune cytokines, and upregulating the expression of immune-related microRNAs. Furthermore, combining BCG-CWS with dengue antigens encapsidated in TMC NPs robustly activated innate antiviral responses. These responses effectively drived an antiviral state of bystander HDFs. Given the unique adjuvant properties of BCG-CWS, it may offer significant advantages over conventional adjuvants for the development of anti-DENV vaccines.

## Supporting information

S1 Fig Cytotoxicity of BCG-CWS-adjuvanted dengue nanovaccine in DCs. MoDCs were incubated with TMC NPs (100 ug/mL), NS1-TMC NPs, or UV-DENV2 TMC NPs (100 µg/mL containing 15 µg/ml of immunogens) in the presence or absence of BCG-CWS (7.5 µg/mL) for 48 h. The 10% DMSO-treated group was the positive control group. The viability of treated cells was determined using the MTS assay. Data are presented as mean ± SD (n = 4).
(TIF)

S1 File Raw data. (XLSX)
