# Supplementary figures and images for: BCG cell wall skeleton augments the immunogenicity of dengue nanoparticle vaccines by promoting dendritic cell activation
Source: PLoS One. 2025 Nov 20;20(11):e0337113. doi: 10.1371/journal.pone.0337113 (PMC12633950; doi:10.1371/journal.pone.0337113)

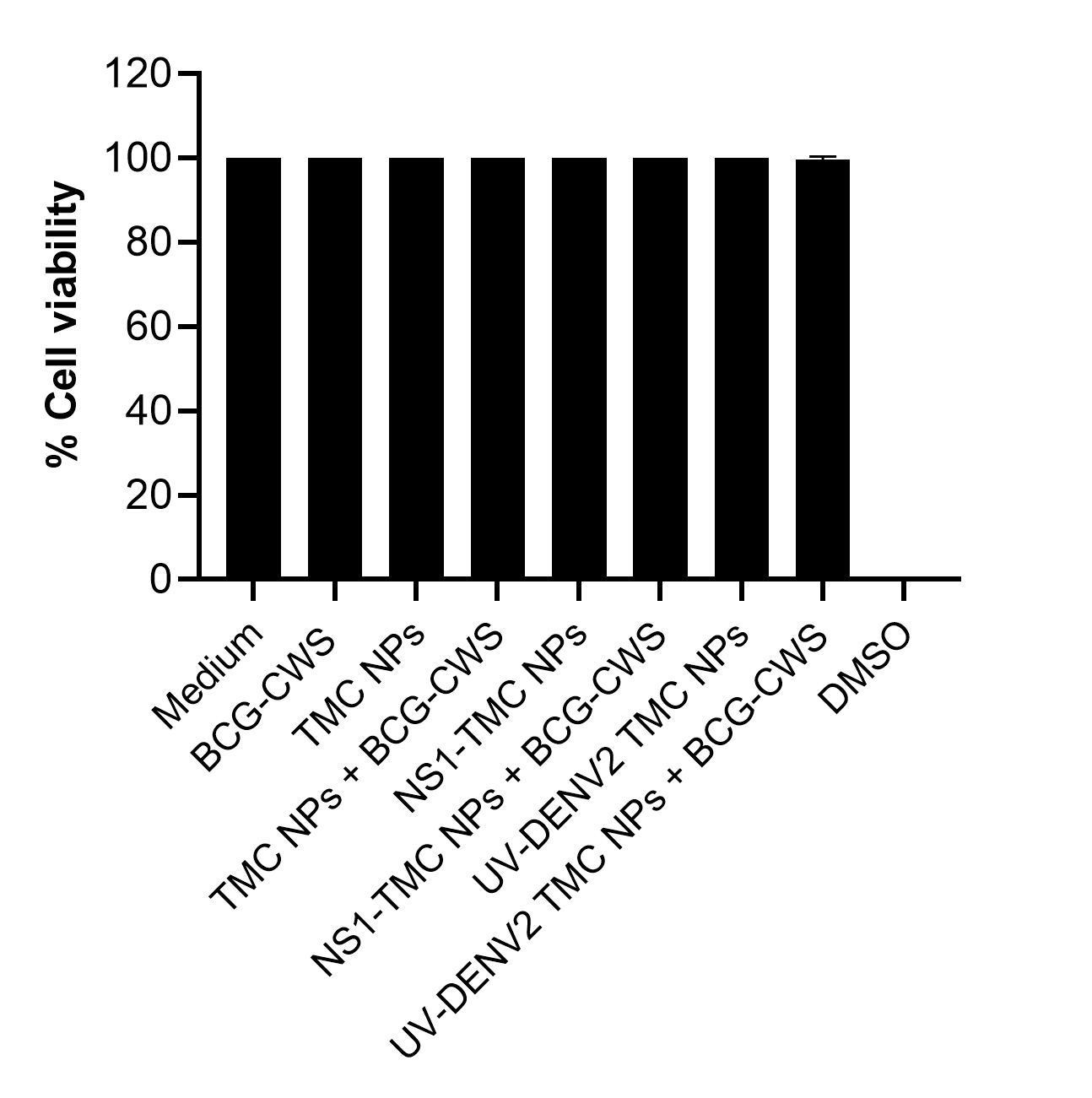

Supplement: S1 Fig — MoDCs were incubated with TMC NPs (100 ug/mL), NS1-TMC NPs, or UV-DENV2 TMC NPs (100 µg/mL containing 15 µg/ml of immunogens) in the presence or absence of BCG-CWS (7.5 µg/mL) for 48 h. The 10% DMSO-treated group was the positive control group. The viability of treated cells was determined using the MTS assay. Data are presented as mean ± SD (n = 4). (TIF) [file pone.0337113.s001.tif]
